# Monocytes acquire prostate cancer specific chromatin conformations upon indirect co-culture with prostate cancer cells

**DOI:** 10.3389/fonc.2022.990842

**Published:** 2022-08-19

**Authors:** Heba Alshaker, Ewan Hunter, Matthew Salter, Aroul Ramadass, Willem Westra, Mathias Winkler, Jayne Green, Alexandre Akoulitchev, Dmitri Pchejetski

**Affiliations:** ^1^ School of Medicine, University of East Anglia, Norwich, United Kingdom; ^2^ Oxford BioDynamics Limited, Oxford, United Kingdom; ^3^ Department of Surgery and Cancer, Imperial College London, London, United Kingdom

**Keywords:** prostate cancer, nucleome, 3C, blood test, horizontal gene transfer

## Abstract

**Background:**

Three-dimensional chromosome loop conformations are powerful regulators of gene expression. These chromosome conformations can be detected both in tumour and in circulating cells and have significant disease biomarker potential. We have recently detected specific chromosome conformations in circulating cells of patients with prostate cancer (PCa) which were similar to ones found in their primary tumours, however, the possibility of horizontal transfer of chromosome conformations was not studied previously.

**Methods:**

Human monocytes (U937) were co-cultured in Boyden chambers through 0.4 uM membrane with or without PC-3 human PCa cells or their conditioned media and a custom DNA microarray for 900,000 chromosomal loops covering all coding loci and non-coding RNA genes was performed on each part of the co-culture system.

**Results:**

We have detected 684 PC-3 cell-specific chromosome conformations across the whole genome that were absent in naïve monocytes but appeared in monocytes co-cultured with PC-3 cells or with PC-3-conditioned media. Comparing PC3-specific conformations to the ones we have previously detected in systemic circulation of high-risk PCa patients revealed 9 positive loops present in both settings.

**Conclusions:**

Our results demonstrate for the first time a proof of concept for horizontal transfer of chromosome conformations without direct cell-cell contact. This carries high clinical relevance as we have previously observed chromatin conformations in circulating cells of patients with melanoma and PCa similar to ones in their primary tumours. These changes can be used as highly specific biomarkers for diagnosis and prognosis. Further studies are required to elucidate the specific mechanism of chromosome conformations transfer and its clinical significance in particular diseases.

## Introduction

Genome-wide association studies have shown that surprisingly the majority of cancer-risk associated loci are located outside of known protein-coding regions (1). It is now well established that epigenetic modifications (DNA methylation, histone acetylation and chromosome conformation) have an important role in aberrant gene expression and cancer progression. In prostate cancer (PCa) DNA methylation (hypo- and hypermethylation) is the best-characterized epigenetic alteration (2, 3). Histone modifications also contribute to PCa progression (4, 5), but the role of chromosome conformations is much less studied.

**Figure 1:**
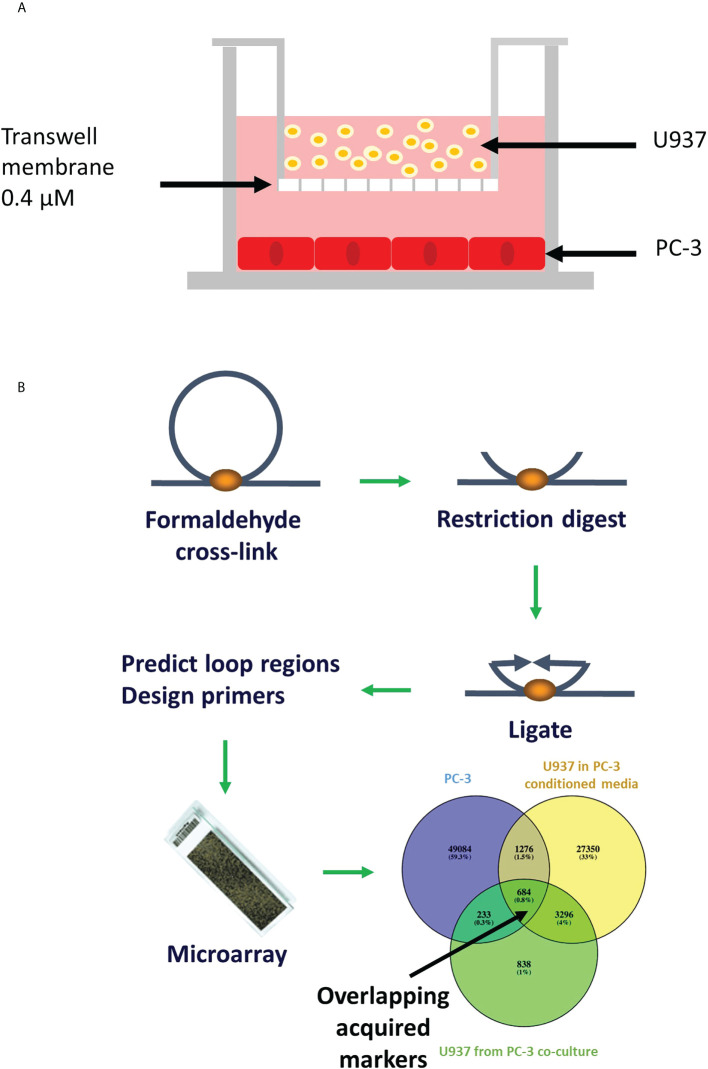
Schematic representation of the transwell model and chromosomal conformation assay. **(A)** PC-3 cells were cultured at the bottom chamber for 48 hours, then U937 cells were placed in the top chamber and incubated for 24 hours. **(B)** Chromosome conformation assay: DNA is crosslinked, digested, ligated and new sequences (in places where loops were) are predicted using relevance machine vector. Loops presence is detected using DNA microarray (11).

Our recent studies have shown significant involvement of 3D chromosome loop interactions in gene expression (6). These dynamic loops can be detected using chromosome conformation capture (3C) technologies. Due to their apparent prevalence in disease, they have gained considerable attention as potential diagnostic markers (7–10). One of the main advantages of 3C-based chromatin interactions as biomarkers is that DNA cross-linking is relatively stable, and following proximity ligation, gives rise to a stable DNA product (**Figure 1**) (12).

We have developed a novel epigenetic assay, as a next generation of the 3C technique (13). This new technology (EpiSwitch™) combines the 3C technique and algorithmic-based analysis to predict and then identify a panel of stable 3D chromosome loops. As these loops are often implicated in aberrant gene expression this technique has significant biomarker potential for the majority of genetic diseases (6). One example of using this technique is distinguishing between cancer-free controls and diseased tissue samples. Using EpiSwitch™ technology, we have detected melanoma-specific chromatin conformations in the circulating cells of melanoma patients (14, 15). A similar subset of conformations were detected in the primary tumours indicating a possible horizontal transfer of epigenetic information (14, 15). In addition to melanoma, we have used this technique to identify specific circulating chromosome conformation signatures (CCSs) for amyotropic lateral sclerosis (16), rheumatoid arthritis (17), and thyroid cancer (18).

Recently we have published distinct CCSs that were present both in circulating cells and primary tumours of PCa patients (11). These signatures allowed both PCa diagnosis and prognostic risk stratification. Similarly to melanoma (14, 15), conformations detected in circulation overlapped with those in PCa tumours suggesting a horizontal transfer (11). Fractionation studies showed that these conformations were coming from circulating white cells and not from circulating tumour cells (14, 15). It remained unclear how circulating cells acquire disease-specific chromosome signatures. In this study, we have used indirect co-culture systems to address a fundamental question of the possibility of the horizontal transfer of epigenetic information in the form of CCSs from primary tumours to circulating blood cells.

## Methods

### Cell culture

Human prostate cancer cell line PC-3 and human monocytes U937 were purchased from ATCC (Manassas, VA, USA). Cells were cultured and maintained in RPMI 1640 Medium, GlutaMAX™ Supplement containing 10% foetal bovine serum and penicillin-streptomycin (5,000 U/mL) (Gibco) at 37°C in a humidified atmosphere of 5% CO_2_. Cell lines were kept in culture for up to 30 passages. For co-cultures, PC-3 cells were seeded into the 6-well plates at 1x10^5^ cells per well in complete medium, as shown in the **Figure 1A**. After 24 hours, media was changed to serum-free for a further 24 hours. Transwells (Corning^®^) containing U937 cells were placed in each well. Cells were harvested separately after 24h co-incubation. For conditioned media experiment, serum-free media incubated with PC-3 cells for 24 hours was collected, centrifuged at 2500rpm for 5 minutes and supernatant added to U937 cells.

### Sample preparation

Whole cell lysate was obtained from individual components of co-culture system by harvesting the cells, centrifuging them 2500rpm for 5 minutes and resuspending them in lysis buffer as described before (11, 14–16). Intrachromatin associations were captured by fixing chromatin with formaldehyde (**Figure 1B**). TaqI restriction enzyme was used for restriction/digestion of chromatin loops into fragments. DNA strands were then rejoined favouring cross-linked fragments. The PCR was performed on reversed crosslinks using the primers previously established by the EpiSwitch™ software (11, 14–16) (**Figure 1B**).

### DNA CHIP analysis

Custom-made CGH Agilent microarray (8x60k) platform was designed to test technical and biological repeats for >900,000 potential chromosome conformations covering all coding loci and non-coding RNA genes. Gene sequences obtained from www.ensembl.org were used for computational prediction of interchromatin interactions using EpiSwitch™ software. Samples generated as described above were hybridized to the array, and differential presence or absence of each chromosome conformation was identified. LIMMA linear modelling with empirical Bayes moderation of the standard errors, subsequent abundance filtering and cluster analysis were used in data analysis as described before (11, 14–16) (**Figure 1B**).

### Nested polymerase chain reaction

Chromosome conformations identified using the DNA CHIP were confirmed using nested PCR performed as recently described (11). Briefly: “Sequence specific oligonucleotides were designed around the chosen sites for screening potential markers by nested PCR using Primer3. All PCR amplified samples were visualized by electrophoresis in the LabChip GX, using the LabChip DNA 1K Version2 kit (Perkin Elmer, Beaconsfield, UK) and internal DNA marker was loaded on the DNA chip according to the manufacturer’s protocol using fluorescent dyes. Fluorescence was detected by laser and electropherogram read-outs translated into a simulated band on gel picture using the instrument software. The threshold we set for a band to be deemed positive was 30 fluorescence units and above.” (11).

## Results

### Identification of the group markers

PC3 cells were cultured alone or with U937 cells *via* membrane (**Figure 1A**) and compared to U937 cells cultured alone or co-cultured with PC3 cells or their conditioned medium. DNA from whole cells was isolated as described in materials and methods and intrachromatin associations were captured using formaldehyde crosslinking, restriction digestion and ligation as described before (11, 14–16) (**Figure 1B**).

A customized CGH Agilent microarray platform (>900k chromosome conformations) was designed to identify chromosome conformations across the whole genome. LIMMA linear modelling with empirical Bayes moderation of the standard errors, subsequent abundance filtering and cluster analysis were used to define the presence or absence of each locus. Nested PCR was used to confirm identified biomarkers.

**Figure 2:**
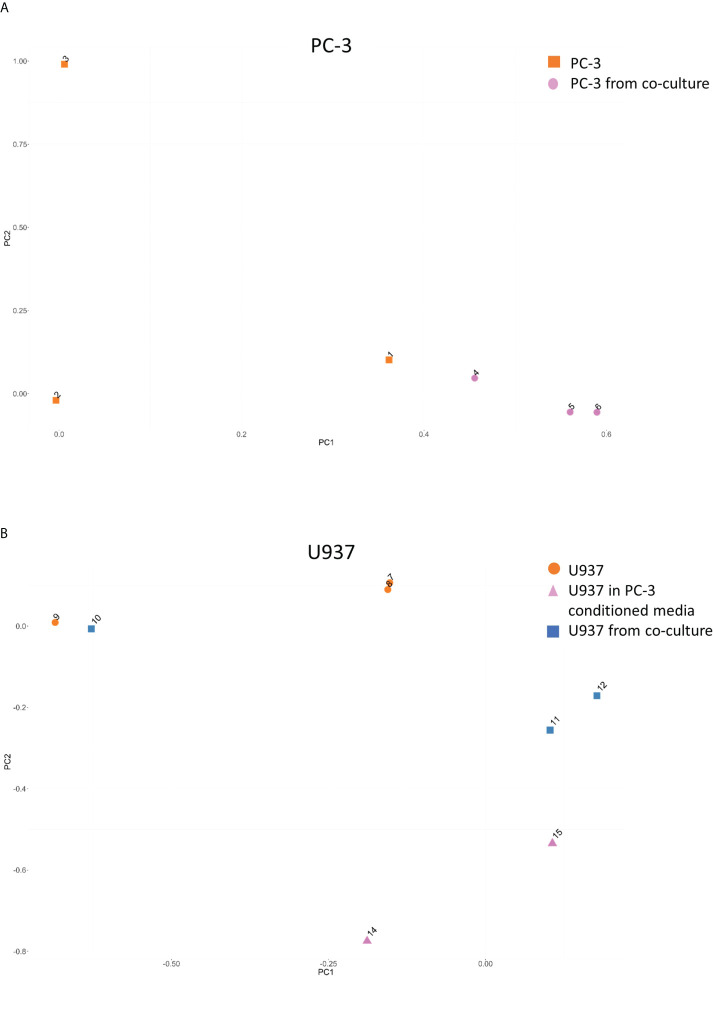
Principal component analysis for the CCSs distribution between samples. Principal component analysis for the CCSs in PC-3 **(A)** and U937 **(B)** before and after co-cultures demonstrating a change in CCSs distribution.

Group variance and presence of outlier samples were assessed using principal component analysis that showed that PC-3 cultured alone (points 1-3 in **Figure 2A**) had a separate 3C profile from PC-3 after co-culture (points 4-6 in **Figure 2A**). Similarly, U937 monocytes showed a clear distinction between cells cultured alone (points 7-9 in **Figure 2B**) and those co-cultured with PC3 cells (points 10-12 in **Figure 2B**) or PC3-conditioned media (points 14,15 in **Figure 2**).

**Figure 3:**
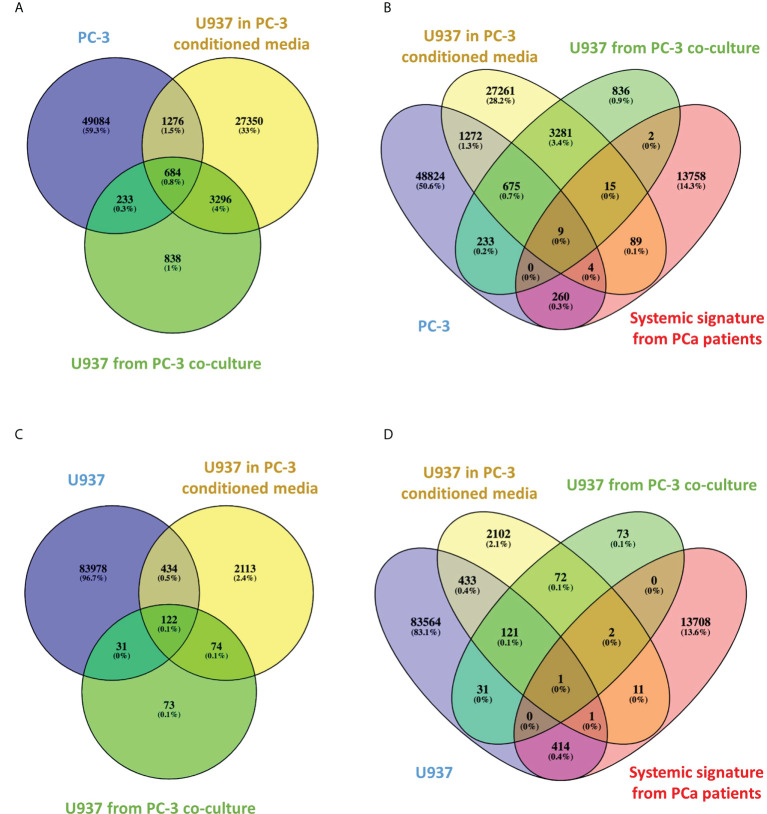
Venn diagrams of CCSs overlap between treatment groups and high-risk PCa patients. **(A–D)**. Venn diagrams indicating the number of overlapping CCSs between various groups.

VENN diagram of PC3-specific CCSs (**Figure 3A**) shows 684 CCSs are shared between PC3 cells and U937 cells cultured with PC-3 cells *via* membrane or in PC3 conditioned media. Each set has interaction frequency over 1.2 and p value ≤0.05. Of note, these statistically significant CCSs are absent in U937 cultured alone. It appears conditioned media induces more CCSs transfer (1960 conformations) than membrane co-culture (917 conformations). Interestingly, functional enrichment analysis of 684 CCSs switching to PC3 profile under both co-culture and conditioned media treatment fit into well-characterized single compact protein interaction network (**Figure S1**). This network has direct relationship to the genetic loci captured by the validated 3C markers that we have identified in the circulating cells of PCa patients (in the loci of *BMP6, ERG, MSR1, MUC1, ACAT1* and *DAPK1* genes) (11) (**Table S1**).

We have then compared the PC3-specific CCSs to the ones we have detected in systemic circulation of high-risk PCa patients (11). The VENN diagram (**Figure 3B**) shows significant (frequency over 1.2 and p value ≤0.05) CCSs present in PC3 and co-cultured monocytes that overlap with systemic PCa array. Nine CCSs are shared between all four sets, whereas many more CCSs are shared between individual sets.

**Table S2** shows the 9 Positive loops present in both high risk PCa patients (11), PC3 cells and monocytes co-cultured with PC3 cells or their conditioned media, but not monocytes alone. Each CCS has three entries tracking up to three nearest coding genes, upstream, downstream and inside the loop, which would be affected by CCS formation. **Table S3** shows the result of pathway analysis for these genes indicating their functional involvement with regulation of prominent cancer and inflammatory pathways such as interleukin (IL)2, epidermal growth factor (EGF), vascular endothelial growth factor (VEGF), p53, tyrosine receptor kinase (TRK)/mitogen activated protein kinase (MAPK), androgen receptor (AR), focal adhesion kinase (FAK), mammalian target of rapamycin (mTOR) and IL6.

Of interest, **Figure 3C** shows 122 CCSs that are present either in naïve U937 cells or in U937 cells co-cultured with PC-3 cells or their conditioned media. Each set has interaction frequency over 1.2, p value ≤0.05 and represents U937 specific CCS, which are not affected by co-culturing. When compared to the systemic PCa signatures identified in our previous study (11), one marker in the *ACAT* loci (**Figure 3D**) was directly shared. In our clinical classification this CCS present only in the U397 cells was a marker of indolent disease.

## Discussion

The CCSs have a well-recognized advantage for the biomarker use (7). This is facilitated by a) the binary nature of the test (the chromosomal loop is either present or not, eliminating diagnostic overlap of continuous biomarkers) and b) the enormous combinatorial power (up to 2^900,000^ combinations are possible with ~900,000 binary loops screened). In conjunction with well-defined clinical groups tested, these features allow creating biomarkers that accurately fit clinical criteria. Indeed, our previous studies demonstrate the significant potential of CCSs as markers for various diseases with different aetiology (11, 14–18). In our recent study in PCa, CCSs allowed us not only to create a diagnostic test, but also a prognostic one, discerning low-risk versus high-risk disease (11). The observed epigenetic changes have been shown to manifest early in tumorigenesis, making them useful for both diagnosis and prognosis (19). It is important to note that CCSs need to be tested in the intact nuclei (13), since the circulating DNA does not retain original 3D conformations.

Our data demonstrate that co-culturing monocytes with PCa cells leads to new stable chromatin loops in the loci of multiple genes (**Figure 3** and **Table S2**) including those we have previously detected in systemic circulation of high-risk PCa patients (11). There is significant evidence supporting their role in human cancers, their prognostic significance and diagnostic value (20–25). Pathway analysis for these genes indicates their functional involvement in the regulation of key pathways involved in cell signalling mediated by cytokines (IL2 and IL6), growth factors (EGF and VEGF), protein kinases (TRK/MAPK, mTOR), tumour suppressors (p53) and hormones (AR) (**Table S3**). All these pathways have high relevance in PCa progression and metastasis (26–28). Using the whole genome array, we have achieved a similar concordance between *ex-vivo* cell models and blood based signatures in our study of diffuse large B-cell lymphoma suggesting that some regulatory aspects of 3D genomics preserve themselves between *ex-vivo* and systemic *in vivo* cellular states (29).

Multiple previous studies have demonstrated horizontal transfer of genetic information (30). In our studies we have shown identical signatures detected in systemic circulation and in the primary site of tumorigenesis (11, 14, 15). Our previous fractionation studies showed that signatures detected in circulation come from white cells and not from circulating tumour cells. We have therefore hypothesised that systemic CCSs may originate in primary tumour upon its direct or indirect contact with the circulating cells. A significant proportion of CCSs are controlled by long non-coding RNAs (31, 32). Previous studies demonstrated that tumour cells secrete non-coding RNAs that are endocytosed by neighbouring or circulating cells and may change their chromosomal conformations (32, 33).

In this study, we have used classical exosome experiment settings (**Figure 1**) to demonstrate how indirect contact between cells (either though a membrane or *via* conditioned media) can mediate this process. In our recent publication we have demonstrated the CCCs that are detected in circulating cells of PCa patients that strongly resemble CCCs detected in primary prostate tumours (11). To replicate such clinical setting in the current mechanistic study we have co-cultured human metastatic hormone refractory PCa cells PC-3 and human monocytes U937. PC-3 is a cell line initiated from a bone metastasis of a grade IV prostatic adenocarcinoma from a 62-year-old man. They represent late stages of PCa. U-937 cell line was derived from malignant cells obtained from the pleural effusion of a male patient with histiocytic lymphoma and immortalised with Epstein-Barr virus (EBV). They are used to study the behaviour and differentiation of monocytes and represent typical human circulating cells.

Throughout the exosome research two of the most important questions pertaining are: a) what is the effector target of exosome traffic and b) what is the mechanism by which exosomes lead to change in phenotype. Our data points that at least partly the exosome traffic targets and switches regulatory 3D architecture in effector cells. That switch is binary, stable and works over the threshold similarly both in cell-cell and cell-conditioned media settings. The switch observed in *in vitro* treatments is consistent with systemic validated switches observed in patients (11).

The results of this study provide mechanistic explanation of the concordance between CCCs detected in circulating cells of PCa patients and in their primary tumours (11). This is likely to pave way to further studies identifying CCCs for PCa diagnosis and screening.

## Conclusions

In this pilot study, reported in this rapid communication, we have identified stable CCSs that are acquired by cells upon indirect co-culture demonstrating for the first-time direct transfer of 3D genome architecture between cancer and circulating cells. These CCSs are similar to the ones we have identified in PCa patients and have significant potential for the development of quick diagnostic and prognostic blood tests for PCa. Future studies are required to address: a) the means of epigenetic information transfer (e.g. exosomes, long non-coding RNA) and b) the potential mechanisms of their effect on the 3D chromosome conformations.

## Data availability statement

The raw data supporting the conclusions of this article will be made available by the authors, without undue reservation.

## Author contributions

EH, AR, AA, and DP conceived the study. HA, EH, MS, AR, AA and DP planned, performed and reviewed experiments and analyzed the data. JG, WW and MW provided critical assessment of the manuscript. All authors participated in writing and editing the manuscript. All authors contributed to the article and approved the submitted version.

## Funding

This work was funded by Oxford BioDynamics.

## Acknowledgments

The authors would like to thank members of Oxford BioDynamics; Benjamin Foulkes, Chloe Bird, Diana Jaramillo Mahecha, Emily Corfield, Warren Elvidge, Ryan Powell for the laboratory work.

## Conflict of interest

EH, MS, AR, WW, JG and AA are employees of Oxford BioDynamics. AA and AR are company directors. Oxford BioDynamics holds patents on the EpiSwitch™ technology.

The remaining authors declare that the research was conducted in the absence of any commercial or financial relationships that could be construed as a potential conflict of interest.

## Publisher’s note

All claims expressed in this article are solely those of the authors and do not necessarily represent those of their affiliated organizations, or those of the publisher, the editors and the reviewers. Any product that may be evaluated in this article, or claim that may be made by its manufacturer, is not guaranteed or endorsed by the publisher.
