# Supplementary material for: Monocytes acquire prostate cancer specific chromatin conformations upon indirect co-culture with prostate cancer cells
Source: Front Oncol. 2022 Aug 19;12:990842. doi: 10.3389/fonc.2022.990842 (PMC9437316; doi:10.3389/fonc.2022.990842)
Supplement: Supplementary file 1 [file DataSheet_1.pdf]

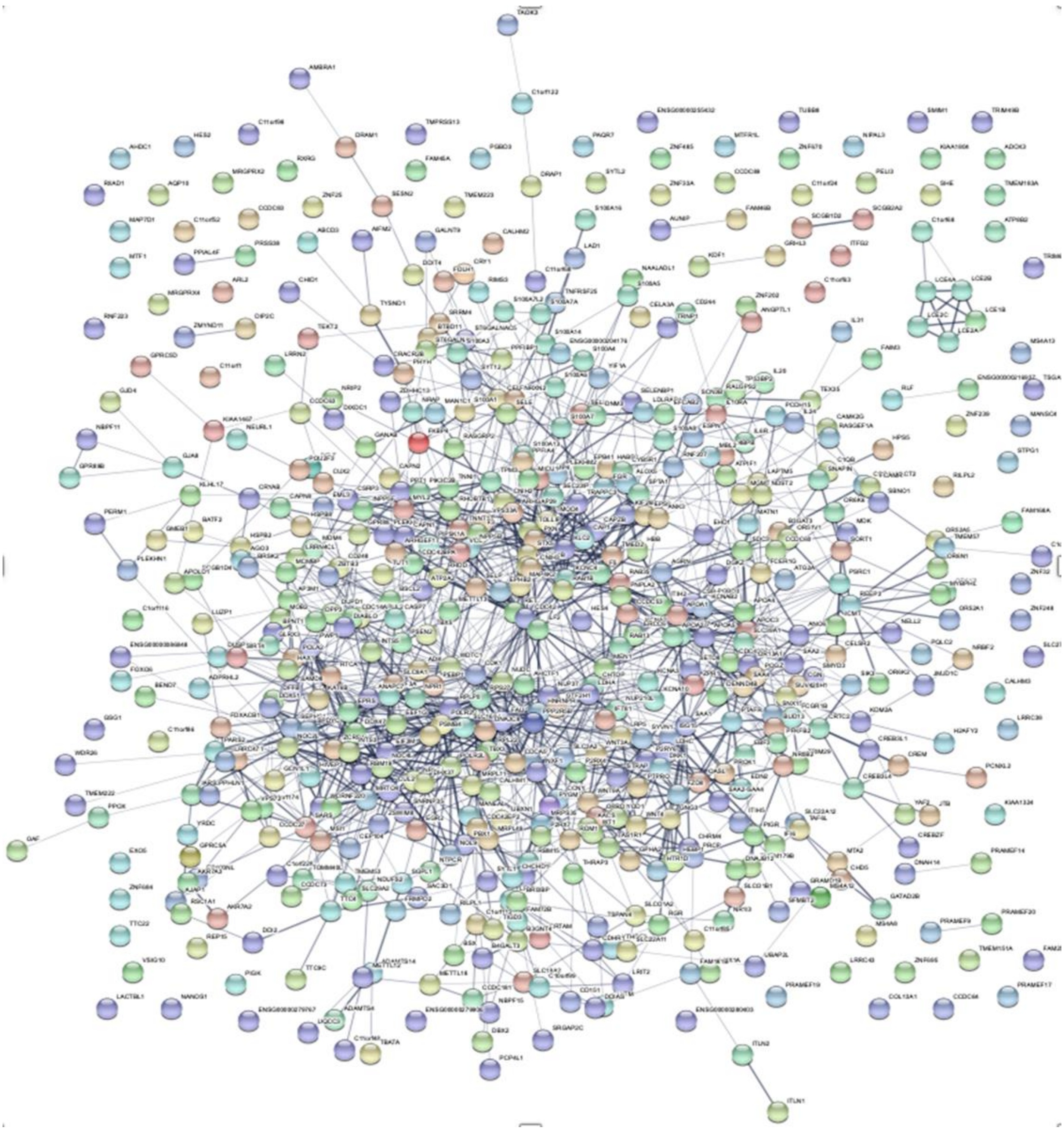

Figure S1. Interaction network for 684 CCS by functional enrichment analysis using String Protein-Protein Interaction Networks (https://string-db.org).

Table S1. Interaction between 684 CCS deregulations in co-culture experiment with genetic loci captured by the validated 3C markers in the circulating cells of high-risk prostate cancer (PCa) patients.

Gene locus from systemic circulation in PCa patients	Interacting genes from co-culture
**BMP6**	WNT3A, WNT9A
**MSR1**	APOA1,2,3,4,5 and APOC3
**DAPK1**	MGMT, TPM3
**MUC1**	AGRN, SELE, ST6ALNAC3, ST6ACNAC5
**ACAT1**	ACAT1 (direct overlap)

Table S2. Nine positive loops present in both high risk PCa patients, PC3 cells and monocytes co-cultured with PC3 cells or their conditioned media, but not monocytes alone. Each CCS has three entries tracking up to three nearest coding genes, upstream, downstream and inside the loop, which would be affected by CCS formation.

1	probes	hg38_probe	Type	Gene	Distance	GeneLocus	P.Value	adj.P.Val	FC_1
2	RCHY1_4_76405310_	Hg38_4_75480100_75487374_75525754_75536935_FF	U937	RCHY1	0	RCHY1	0.00042	0.004998	1.130748
3	RCHY1_4_76405310_	Hg38_4_75480100_75487374_75525754_75536935_FF	U937	THAP6	0	RCHY1	0.00042	0.004998	1.130748
4	RCHY1_4_76405310_	Hg38_4_75480100_75487374_75525754_75536935_FF	U937	C4orf26	19114	RCHY1	0.00042	0.004998	1.130748
5	MDM4_1_204497005	Hg38_1_204527877_204535378_204577840_204585375_RF	Pc3	MDM4	0	MDM4	0.012434	0.046922	1.118609
6	MDM4_1_204497005	Hg38_1_204527877_204535378_204577840_204585375_RF	Pc3	LRRN2	31796	MDM4	0.012434	0.046922	1.118609
7	MDM4_1_204497005	Hg38_1_204527877_204535378_204577840_204585375_RF	Pc3	PIK3C2B	33154	MDM4	0.012434	0.046922	1.118609
8	SUZ12_17_30287449	Hg38_17_31960430_31964626_32012814_32017352_FR	Pc3	SUZ12	0	SUZ12	0.016934	0.058203	-1.05028
9	SUZ12_17_30287449	Hg38_17_31960430_31964626_32012814_32017352_FR	Pc3	SUZ12	0	SUZ12	0.016934	0.058203	-1.05028
10	SUZ12_17_30287449	Hg38_17_31960430_31964626_32012814_32017352_FR	Pc3	LRRC37B	0	SUZ12	0.016934	0.058203	-1.05028
11	SUZ12_17_30287449	Hg38_17_31960430_31964626_32012814_32017352_FR	Pc3	LRRC37B	0	SUZ12	0.016934	0.058203	-1.05028
12	SUZ12_17_30287449	Hg38_17_31960430_31964626_32012814_32017352_FR	Pc3	UTP6	58666	SUZ12	0.016934	0.058203	-1.05028
13	SUZ12_17_30287449	Hg38_17_31960430_31964626_32012814_32017352_FR	Pc3	UTP6	58666	SUZ12	0.016934	0.058203	-1.05028
14	RPS6KB1_17_580138	Hg38_17_59936509_59940264_59960962_59964528_FR	Pc3	RPS6KB1	0	RPS6KB1	0.025874	0.078206	1.087696
15	RPS6KB1_17_580138	Hg38_17_59936509_59940264_59960962_59964528_FR	Pc3	RPS6KB1	0	RPS6KB1	0.025874	0.078206	1.087696
16	RPS6KB1_17_580138	Hg38_17_59936509_59940264_59960962_59964528_FR	Pc3	RPS6KB1	0	RPS6KB1	0.025874	0.078206	1.087696
17	RPS6KB1_17_580138	Hg38_17_59936509_59940264_59960962_59964528_FR	Pc3	RP11-178C	0	RPS6KB1	0.025874	0.078206	1.087696
18	RPS6KB1_17_580138	Hg38_17_59936509_59940264_59960962_59964528_FR	Pc3	RP11-178C	0	RPS6KB1	0.025874	0.078206	1.087696
19	RPS6KB1_17_580138	Hg38_17_59936509_59940264_59960962_59964528_FR	Pc3	RP11-178C	0	RPS6KB1	0.025874	0.078206	1.087696
20	RPS6KB1_17_580138	Hg38_17_59936509_59940264_59960962_59964528_FR	Pc3	RNFT1	0	RPS6KB1	0.025874	0.078206	1.087696
21	RPS6KB1_17_580138	Hg38_17_59936509_59940264_59960962_59964528_FR	Pc3	RNFT1	0	RPS6KB1	0.025874	0.078206	1.087696
22	RPS6KB1_17_580138	Hg38_17_59936509_59940264_59960962_59964528_FR	Pc3	RNFT1	0	RPS6KB1	0.025874	0.078206	1.087696
23	MDM4_1_204497005	Hg38_1_204527877_204535378_204577840_204585375_RR	Pc3	MDM4	0	MDM4	0.028852	0.083965	-1.04773
24	MDM4_1_204497005	Hg38_1_204527877_204535378_204577840_204585375_RR	Pc3	LRRN2	31796	MDM4	0.028852	0.083965	-1.04773
25	MDM4_1_204497005	Hg38_1_204527877_204535378_204577840_204585375_RR	Pc3	PIK3C2B	33154	MDM4	0.028852	0.083965	-1.04773
26	BRAF_7_140549408_	Hg38_7_140849608_140854743_140881474_140888633_FR	Pc3	BRAF	0	BRAF	0.029715	0.08574	-1.05503
27	BRAF_7_140549408_	Hg38_7_140849608_140854743_140881474_140888633_FR	Pc3	MRPS33	113978	BRAF	0.029715	0.08574	-1.05503
28	BRAF_7_140549408_	Hg38_7_140849608_140854743_140881474_140888633_FR	Pc3	NDUFB2	126819	BRAF	0.029715	0.08574	-1.05503

Table S3. Functional pathway analysis for the genes shared between high risk PCa patients acquired by monocytes upon co-culture (https://geneanalytics.genecards.org).

Score	Name	# Matched Genes (Total Genes)	Sources
18.62	SuperPath: Glioma	5 (211)	
18.28	SuperPath: TGF-beta Signaling Pathways	4 (96)	RD
16.73	SuperPath: Crizotinib Pathway, Pharmacokinetics/Pharmacodynamics	3 (38)	
15.56	SuperPath: IL-2 Pathway	5 (328)	
15.26	SuperPath: EGF/EGFR Signaling Pathway	4 (164)	
14.92	SuperPath: VEGF Pathway (Tocris)	3 (58)	
14.84	SuperPath: P53 Pathway	3 (59)	
12.48	SuperPath: CNTF Signaling	3 (103)	
12.35	SuperPath: Serotonin Receptor 4/6/7 and NR3C Signaling	2 (19)	
12.16	SuperPath: Glioblastoma Multiforme	3 (111)	
11.24	SuperPath: IGF1 Pathway	2 (28)	
11.14	SuperPath: Trk Receptor Signaling Mediated By The MAPK Pathway	2 (29)	
11.13	SuperPath: Common Cytokine Receptor Gamma-Chain Family Signaling Pathways	3 (142)	RD
10.77	SuperPath: BDNF-TrkB Signaling	2 (33)	
10.65	SuperPath: Prolactin Signaling Pathway	3 (159)	
10.30	SuperPath: 4-1BB Pathway	3 (173)	
9.59	SuperPath: Regulation of Androgen Receptor Activity	2 (50)	
9.42	SuperPath: G-AlphaQ Signaling	3 (214)	
9.37	SuperPath: Signaling Events Mediated By Focal Adhesion Kinase	2 (54)	
9.07	SuperPath: MTOR Signaling Pathway (Pathway Interaction Database)	2 (60)	
8.93	SuperPath: IL-6 Signaling Pathway	2 (63)	
8.72	SuperPath: Human Thyroid Stimulating Hormone (TSH) Signaling Pathway	2 (68)	
8.48	SuperPath: Translation Translation Regulation By Alpha-1 Adrenergic Receptors	2 (74)	
